# Dysregulated myokines and signaling pathways in skeletal muscle dysfunction in a cigarette smoke–induced model of chronic obstructive pulmonary disease

**DOI:** 10.3389/fphys.2022.929926

**Published:** 2022-08-25

**Authors:** Lijiao Zhang, Chunxiao Li, Jing Xiong, Chun Chang, Yongchang Sun

**Affiliations:** ^1^ Department of Respiratory and Critical Care Medicine, Peking University Third Hospital, Beijing, China; ^2^ Department of Radiation Oncology, Peking University Third Hospital, Beijing, China

**Keywords:** chronic obstructive pulmonary disease (COPD), cigarette smoke, FNDC5/irisin, myostatin, ERK, skeletal muscle dysfunction

## Abstract

Skeletal muscle dysfunction is an important extrapulmonary comorbidity of chronic obstructive pulmonary disease (COPD). Muscle-derived cytokines (myokines) play important roles in skeletal muscle growth and function, but their contributions to skeletal muscle dysfunction in COPD have not been fully understood. In the current study, by using a well-established mouse model of COPD with skeletal muscle dysfunction, we found that the expressions of Fndc5 (fibronectin type III domain-containing protein 5, the precursor of irisin) and peroxisome proliferator-activated receptor-γ coactivator 1α (PGC-1α) were decreased, while myostatin (Mstn), phosphorylated extracellular regulated kinase (p-Erk1/2), and p-Smad3 expressions were upregulated in skeletal muscles from cigarette smoke-exposed mice and in cigarette smoke extract (CSE)-stimulated C2C12 myotubes. Treatment with Smad3 or Erk1/2 inhibitors partially restored the expression of Fndc5 in CSE-stimulated C2C12 myotubes. Taken together, CSE exposure, by upregulation of p-Erk1/2, promoted the expression of Mstn, which further inhibited Fndc5 expression by the p-Smad3/PGC-1α pathway, revealing a novel regulating mechanism of myokines in the pathogenesis of skeletal muscle comorbidities of COPD.

## Introduction

Chronic obstructive pulmonary disease (COPD) is a globally prevalent chronic airway disease associated with exposure to noxious particles or gases, especially cigarette smoke (CS), which leads to persistent airway inflammation and lung destruction (emphysema) (Vogelmeier et al., 2021). COPD is now considered as a systemic disease with multiple extrapulmonary comorbidities, such as cardiovascular diseases, metabolic syndrome, skeletal muscle dysfunction/sarcopenia, and osteoporosis (Hillas et al., 2015), which have a significant impact on morbidity and mortality of COPD. The estimated prevalence of sarcopenia in COPD patients was 27.5% by a meta-analysis (Sepúlveda-Loyola et al., 2020). We find it interesting that skeletal muscle dysfunction/sarcopenia is more significant in individuals with emphysema and lower diffusing capacity, than in those with airway-type COPD (Vanfleteren et al., 2013), and is associated with increased symptom burden and poorer prognosis of the disease (Byun et al., 2017; Jaitovich and Barreiro, 2018). Skeletal muscle dysfunction/sarcopenia impairs ambulatory capacity, which in turn contributes to muscle disuse and atrophy, forming a vicious circle.

The risk factors underlying skeletal muscle dysfunction in COPD are varied, including systemic inflammation, systemic administration of corticosteroids, advanced age, malnutrition, hypercapnia, hypoxia, deconditioning, and oxidative stress (Yende et al., 2006; Barreiro et al., 2015). Skeletal muscle dysfunction in COPD is a multifactorial and multistep process. Muscle proteolysis involves complicated cellular networks, in which the ubiquitin-proteasome pathway predominates (Plant et al., 2010). Two pivotal muscle-specific E3-ubiquitin ligases, muscle-specific ring finger 1 (MuRF1, also known as TRIM63) and Atrogin1 (also known as FBXO32), are upregulated in muscle atrophy, and loss of either can reduce the rate of muscle atrophy (Cohen et al., 2015). In addition, autophagy and apoptosis may be also involved in the muscle dysfunction of COPD (Jaitovich and Barreiro, 2018).

Skeletal muscle has been identified as a secretory organ in the past decade (Pedersen and Febbraio, 2012), in addition to its critical role as an essential component of the motion system. Irisin is a peroxisome proliferator-activated receptor-γ coactivator 1α (PGC-1α)-dependent myokine mainly derived from skeletal muscles (Boström et al., 2012) and is regulated by exercise (Daskalopoulou et al., 2014; Perakakis et al., 2017). PGC-1α, a transcriptional coactivator, is induced by exercise in muscles and mediates a series of beneficial effects of exercise, such as mitochondrial biogenesis, angiogenesis, and fiber-type switching (Handschin and Spiegelman, 2008). Studies have confirmed that the transforming growth factor β (TGF-β)/SMAD3 signals negatively regulate the expression and/or secretion of irisin via suppressing the expression of the irisin precursor Fndc5 (fibronectin type III domain-containing protein 5) and its upstream activator, PGC-1α, in skeletal muscle cells (Tiano et al., 2015). Previous studies have found dysregulated serum irisin in COPD, suggesting that irisin may be involved in the pathogenesis of COPD and its skeletal muscle comorbidity (Greulich et al., 2014; Ijiri et al., 2015; Boeselt et al., 2017; Sugiyama et al., 2017; Kubo et al., 2019; Zhang and Sun, 2021). However, the expression of Fndc5 and its regulation mechanisms in COPD are still unknown.

Myostatin (Mstn), another well-known myokine, belongs to the TGF-β superfamily. Mstn is primarily derived from skeletal muscles and serves as a potent inhibitor of muscle growth (McPherron et al., 1997). As the most important risk factor for COPD, cigarette smoking could impair muscle protein synthesis and increase the expression of Mstn and MAFbx in the smoker’s skeletal muscles (Petersen et al., 2007). Studies have also shown that Mstn is upregulated and involved in COPD-related skeletal muscle dysfunction (Plant et al., 2010; Polkey et al., 2019; Sancho-Muñoz et al., 2021); however, the molecular mechanisms regulating the expression of Mstn, and particularly its interaction with irisin, in the pathogenesis of skeletal muscle dysfunction in COPD have not yet been elucidated. Therefore, by using a well-established mouse model of COPD induced by long-term CS exposure, and an *in vitro* cell culture model, we screened dysregulated genes and particularly investigated the expressions of Fndc5, Mstn, and related signaling molecules associated with skeletal muscle dysfunction in COPD.

## Materials and methods

### Cigarette smoke–induced mouse model of chronic obstructive pulmonary disease

All animal experiment protocols were in accordance with the committee’s animal care and use guidelines and were approved by the Animal Care Committee of Peking University (No. LA2021545). Aged-matched female C57BL/6 mice (6–8 weeks old) were purchased from Beijing Vital River Laboratory or Charles Rivers Laboratories. All mice were specific-pathogen-free animals and housed in a barrier environment under a 12-h:12-h light/dark cycle. The mouse model of COPD was established using a nose-only cigarette smoking exposure method as reported previously (Xiong et al., 2021). In our previous studies, a mouse model of COPD has been established successfully with the commercial Bai Sha cigarettes (Zhou et al., 2019; Xiong et al., 2020a; Xiong et al., 2020b; Rao et al., 2021; Xiong et al., 2021). In order to exclude the effect of batch variation of cigarettes, the same batch of cigarettes for experiments has been used here. To put it briefly, mice have been simultaneously exposed to commercial cigarettes [Baisha cigarettes with filter, Hunan, China (tar 10 mg, nicotine 0.9 mg, CO 12 mg)] twice/day for 50 min each time, with 20-min smoke-free intervals, 5 days a week, for 24 weeks using a nose-only smoke exposure system (SG-300; SIBATA, Tokyo, Japan). Cigarettes were actively burned, and smoke was generated by a computer-controlled suction, with 20 ml smoke per 8 s into the exposure chamber. The control mice were exposed to filtered air by using the same protocol.

### Grip force test and histology analysis of skeletal muscles

After 24-week CS exposure, the grip force test was performed to evaluate the muscle strength of limbs using a grip strength meter (unibiolab, Beijing, China) as reported previously (Kehl et al., 2000). The researchers were blinded to group allocation. In brief, the mouse was grasping a mesh bar connected to a force transducer with all four limbs and was being pulled back by the tail at a constant speed until its grip was released. The peak force (g) produced during the process was recorded using the transducer. The mean of three attempts was recorded as grip strength. The cross-sectional area (CSA) of muscle fibers was measured as described previously (Chiu et al., 2018). Transversal gastrocnemius muscle sections (10 µm thick) were cut from paraffin-embedded muscle tissues using a cryostat (Leica, Solms, Germany) and stained with hematoxylin–eosin (H&E). Muscle sections were photographed and tiled using the Leica CTR 6500 microscope equipped with the Leica DFC 420 camera and Image Pro Plus software (Media Cybernetics, Maryland, United States). The CSA of muscle fibers was analyzed (*n* ≥ 3 mice per group).

### Histology analysis of mouse lungs

To evaluate pulmonary emphysema, we used mean linear intercept (MLI) and destructive index (DI) to measure the airspace enlargement and destruction of alveolar walls, respectively, following our previous protocol (Zhou et al., 2019). The researchers were also blinded to group allocation. MLI was measured by using a 100 × 100 μm grid passing randomly through the lung and calculated as the total length of each line of the grid divided by the number of alveolar intercepts. The measurement of DI was performed by a grid with 42 points, which were at the center of hairline crosses superimposed on the lung field. Structures lying under these points were marked as normal (N) or destroyed (D) alveolar and/or duct spaces. Points falling over other structures, such as duct walls and alveolar walls, did not enter into the calculations. DI was calculated as D/(D + N). We also estimated the extent of lung inflammatory infiltration by an inflammation score (Zhou et al., 2015). In brief, the degree of peribronchial inflammation was evaluated on a scale of 0–4, with 0 indicating no inflammatory cells detectable, 1 indicating occasional cuffing with inflammatory cells, 2 indicating most bronchi surrounded by a ring of inflammatory cells that were one cell layer deep, 3 indicating most bronchi surrounded by a ring of inflammatory cells that were two to four cells deep, and 4 indicating most bronchi surrounded by a thick layer of inflammatory cells that were over four cells deep.

### Hematoxylin–eosin, immunofluorescence stainings, and immunohistochemistry

The muscle tissues were fixed in 4% polyoxymethylene and then embedded in paraffin. H&E staining procedures were performed as described previously (Feldman and Wolfe, 2014). For immunofluorescence (IF) and immunohistochemistry (IHC) staining, 5-µm-thick paraffin-embedded muscle sections were processed using a standard histological protocol. The paraffin slides were deparaffinized, rehydrated through graded alcohols to deionized water, and subjected to antigen retrieval in a pressure cooker, at 100°C for 20 min. Blocking was performed using Dako protein Block, 3% BSA (Merck Millipore, Massachusetts, Germany) in TBST, and 5% goat serum in TBST. The primary antibodies, including anti-FNDC5 antibody (bs-8486R, Bioss, Beijing, China) (1:1,000), anti-GDF8/myostatin antibody (ab203076, Abcam, Cambridge, United Kingdom) (1:1,000), antimyosin heavy chain (MyHC)-IID antibody (bs-5885R, Bioss, Beijing, China) (1:1,000), and anti-MYH7 (MyHC (slow)) antibody (GB111857, Servicebio, Wuhan, China) (1:1,000), were incubated with the tissue slides overnight at 4°C. Slides were washed in TBST and incubated with secondary antibodies (ab150115, ab150077, and ab6721, Abcam, Cambridge, United Kingdom) diluted in 5% skimmed milk in TBST for 1 h at room temperature. Confocal microscopy was applied to evaluate the expression of FNDC5 and Mstn.

### Total RNA extraction for RNA sequencing

Total RNA was extracted from the muscle tissues using Trizol (Invitrogen, Carlsbad, California, United States) according to the manual instruction. Approximately 60 mg of tissues was ground into powder using liquid nitrogen in a 2 ml tube, followed by homogenization for 2 min and rested horizontally for 5 min. The mix was centrifuged at 12,000×*g* for 5 min at 4°C, and then the supernatant was transferred into a new EP tube with 0.3 ml chloroform/isoamyl alcohol (24:1). The mix was shaken vigorously for 15 s and then centrifuged at 12,000×*g* for 10 min at 4°C. After centrifugation, the superior aqueous phase in which the RNA remained was transferred into a new tube with an equal volume of isopropyl alcohol supernatant and then centrifuged at 13,600 rpm for 20 min at 4°C. After discarding the supernatant, the RNA pellet was washed twice with 1 ml 75% ethanol, and then the mix was centrifuged at 13,600 rpm for 3 min at 4°C to collect residual ethanol, followed by the pellet being air-dried for 5–10 min in the biosafety cabinet. Final, 25–100 μl of DEPC-treated water was added to dissolve the RNA. Next, the total RNA was qualified and quantified through a NanoDrop and Agilent 2100 bioanalyzer (Thermo Fisher Scientific, MA, United States).

### mRNA library construction

Oligo (dT)-attached magnetic beads were used to purify mRNA. The purified mRNA was fragmented into small pieces with a fragment buffer at the proper temperature. Then, first-strand cDNA was generated using random hexamer-primed reverse transcription, followed by a second-strand cDNA synthesis. Afterward, A-Tailing Mix and RNA Index adapters were added by incubation until final repair. The cDNA fragments obtained from the previous step were amplified by PCR, and products were purified by Ampure XP Beads and then dissolved in EB solution. The product was validated on the Agilent Technologies 2100 bioanalyzer for quality control purposes. The double-stranded PCR products from the previous step were denatured by heat and circularized by the splint oligo sequence to get the final library. Single-strand DNA (ssCir DNA) was formatted as a final library. The final library was amplified with phi29 to make a DNA nanoball (DNB) that had more than 300 copies of one molecule; DNBs were loaded into the patterned nanoarray, and single end 50 bases reads were generated on the BGIseq500 platform (BGI-Shenzhen, China).

### RNA-seq and bioinformatic analysis

The sequencing data were filtered using SOAPnuke (v1.5.2) (Li et al., 2008) by 1) removing reads containing sequencing adapter; 2) removing reads whose low-quality base ratio (base quality less than or equal to 5) was more than 20%; and 3) removing readings with an unknown base report (“N”) greater than 5%, and then clean readings were obtained and stored in FASTQ format. The clean reads were mapped to the reference genome using HISAT2 (v2.0.4) (Kim et al., 2015). Bowtie2 (v2.2.5) (Langmead and Salzberg, 2012) was applied to align the clean reads to the reference coding gene set, and then the expression level of gene was calculated using RSEM (v1.2.12) (Li and Dewey, 2011). The heatmap was drawn using pheatmap (v1.0.8) according to the gene expression in different samples. In essence, differential expression analysis was performed using DESeq2 (v1.4.5) with a Q value ≤0.05. To take insight to the change of phenotype, GO (http://www.geneontology.org/) and KEGG (https://www.kegg.jp/) enrichment analyses of annotated differentially expressed genes was performed using Phyper (https://en.wikipedia.org/wiki/Hypergeometric_distribution) based on the hypergeometric test. Significant levels of terms and tracks were corrected using the Q value with a stringent threshold (Q value 0.05) using the Bonferroni test.

### Cigarette smoke extract preparation

Cigarette smoke extract (CSE) was prepared as previously described (Gellner et al., 2016). CSE was freshly prepared by bubbling the smoke from five cigarettes (Baisha, Hunan, China; tar 10 mg, nicotine 0.9 mg, CO 12 mg) through 10 ml of serum-free prewarmed (37°C) cell culture medium. Five cigarettes were smoked at a constant airflow, lasting 5 min for each cigarette. After adjustment of the pH to 7.4, the obtained extract was filtered through a 0.22-μm pore filter (Merk Millipore, Massachusetts, Germany). To ensure a similar preparation among different batches, we used a spectrophotometer to measure the absorbance at 320 nm. The mean optical density was 4.0, and this solution was considered 100%. CSE was utilized within 30 min after preparation.

### Cell culture and myotube formation

The mouse C2C12 myoblasts (American Type Culture Collection, Manassas, United States) were cultured in Dulbecco’s modified Eagle’s medium (DMEM) (Thermofisher, Massachusetts, United States) supplemented with 10% fetal bovine serum (Thermofisher, Massachusetts, United States), 2 mM glutamine, 100 IU/ml penicillin, and 100 mg/ml streptomycin (Merk Millipore, Germany) at 37°C with saturated humidity and 5% carbon dioxide. Trypsin-EDTA was used to detach cells from tissue culture dishes. The dissociated C2C12 myoblasts were subcultured in a new dish. When the myoblasts grew to 70–80% confluence, the culture medium was switched to differentiation medium consisting of DMEM containing 2% horse serum (Thermofisher, Massachusetts, United States) for 5 days to induce myoblasts to differentiate into myotubes. In this study, C2C12 myotubes were treated with drugs, including PGC-1α activator ZLN005 (MedChemExpress, New Jersery, United States), Human/Murine/Rat Irisin (Pepro Tech, New Jersery, United States), Recombinant Human/Murine/Rat Myostatin (Pepro Tech, New Jersery, United States), activin-like kinase (ALK) 4/5 inhibitor TEW-7197 (Selleck, Shanghai, China), Smad3 inhibitor SIS3 (Selleck, Shanghai, China), ERK inhibitor U0126 (Selleck, Shanghai, China), and a vehicle (dimethylsufoxide, DMSO).

### Cell viability by cell counting kit-8 assay

Cell viability was tested using the cell counting kit-8 (CCK-8) assay according to the manufacturer’s instructions. Our previous studies had shown that the concentration of CSE lower than 3% had no effects on cellular viability (Xiong et al., 2021), and we verified the effect of CSE on cell viability with concentration gradients of CSE. C2C12 myotubes were seeded at a density of 10,000 cells/well in 96-well plates and stimulated with CSE concentration of 0, 0.5%, 1.0%, 1.5%, 2.0%, 2.5%, 3.0%, 3.5%, 4.0%, 4.5%, 5.0%. and 10%. The cells were incubated under conditions with saturated humidity and 5% carbon dioxide at 37°C overnight. Then, 10 μl of CCK-8 solution (Solarbio, Beijing, China) was added into each well, and the cells were incubated for another 4 h. Last, the absorbance was measured at 450 nm using a spectrophotometer. The C2C12 myotubes were treated by 3%, 1.5%, and 0.75% CSE with indicated time.

### Flow cytometry

The differentiated C2C12 myotubes were stimulated with CSE (3%, 1.5%, and 0.75%) for 24 and 48 h. Before harvest, cells were treated with Monensin (BioLegend, California, United States) for another 4–6 h. Then, cells were incubated with fluorescence-conjugated antibodies, including rabbit antimouse myostatin antibody (ab203076, Abcam, Cambridge, United Kingdom), rabbit antimouse FNDC5 antibody (bs-8486R, Bioss, Beijing, China), antimouse PGC-1α antibody (AF7736, Beyotime, Shanghai, China), and antimouse MuRF1 antibody (bs-2539R, Bioss, Beijing, China). The second antibodies included PE antirabbit IgG (12-4739-81, eBioscience, United States) and isotype PE antirabbit IgG (400707, BioLegend, United States). The cells were then fixed in 0.5 ml/tube Fixation Buffer (BioLegend) in the dark for 20 min at room temperature. Permeabilization was performed using Intracellular Staining Perm Wash Buffer (BioLegend). Flow cytometry data were acquired on a CytoFlex LX flow cytometer (Beckman Coulter, CA, United States) and analyzed using FlowJo (Tree Star, San Carlos, California, United States).

### RNA extraction, reverse transcription, and quantitative PCR

Total RNA was extracted from the gastrocnemius muscle of mice and C2C12 myotubes using Trizol (Invitrogen, Carlsbad, California, United States) following manufacturer’s instruction and then reverse transcribed into complementary DNA (cDNA) with PrimeScript RT Master Mix (TAKARA biotechnology, Dalian, China). cDNA was quantified using SYBR Green real-time PCR with 500 nM primers using QuantStudio™ 5 (Thermo Fisher). *Gapdh* was used as the reference gene. The primer sequences used for the amplification were as follows: *Gapdh* (forward) 5′-TCA​ACG​ACC​ACT​TTG​TCA​AGC​TCA-3′, (reverse) 5′-GCT​GGT​GGT​CCA​GGG​GTC​TTA​CT-3′; *Fndc5* (forward) 5′-TTG​CCA​TCT​CTC​AGC​AGA​AGA-3′, (reverse) 5′-GGC​CTG​CAC​ATG​GAC​GAT​A-3′; *Mstn* (forward) 5′-AGT​GGA​TCT​AAA​TGA​GGG​CAG​T-3′, (reverse) 5′-GTT​TCC​AGG​CGC​AGC​TTA​C-3′; and *MuRF1* (forward) 5′-GTG​TGA​GGT​GCC​TAC​TTG​CTC-3′, (reverse) 5′-GCT​CAG​TCT​TCT​GTC​CTT​GGA-3′. The relative gene expression to control was calculated using the following formula: 2^−ΔΔCT^ = 2_sample_^−ΔCT^/2_Ctrl_^−ΔCT^.

### Western blotting

Muscle tissues and cells were homogenized in RIPA buffer (containing 50 mM pH 7.4 Tris–HCl, 1% Triton X-100, 150 mM NaCl, 0.1% SDS, 1% sodium deoxycholate, protease, and phosphatase inhibitor cocktail) on ice and then centrifuged at 1,000 *g* for 10 min at 4°C. The supernatant was stored at −80°C. Protein concentration was measured using the BCA method. Approximately 30 μg protein of each samples was loaded in on 10% SDS–PAGE and run at 90–120 V constant voltage. A constant current of 300 mA was used for transblotting with 0.45 μm PVDF (Merk Millipore, Germany). Blots were probed with primary antibodies, including antimouse GAPDH antibody (sc-47724, Santa Cruz Biotechnology, Texas, United States) (1:1,000), antiphosphorylated Smad3 antibody (AF1759, Beyotime, Shanghai, China) (1:1,000), anti-Smad3 antibody (ab40854, Abcam, Cambridge, United Kingdom) (1:1,000), anti-PGC-1α antibody (AF7736, Beyotime, Shanghai, China) (1:1000), anti-GDF8/myostatin antibody (ab203076, Abcam, Cambridge, United Kingdom) (1:1,000), anti-FNDC5 antibody (bs-8486R, Bioss, Beijing, China) (1:1000), antiphosphorylated Erk1/2 antibody (4370, CST, United States) (1:1000), anti-Erk1/2 antibody (4695, CST, United States) (1:1,000), anti-MuRF1 antibody (bs-2539R, Bioss, Beijing, China) (1:1,000), anti-FBXO32 antibody (bsm-54451R, Bioss, Beijing, China) (1:1,000), anti-MyHC-IID antibody (bs-5885R, Bioss, Beijing, China) (1:1,000), and anti-MYH7 (MyHC (slow)) antibody (GB111857, Servicebio, Wuhan, China) (1:1,000), overnight at 4°C. After washing five times, blots were then incubated with goat antirabbit or goat antimouse secondary antibody (ab205718 and ab6721, Abcam, Cambridge, United Kingdom) (1:2,000) at room temperature for 2 h. Chemiluminescence was used to visualize protein bands using Immobilon Western Chemiluminescent HRP Substrate (WBKLS0500, Merk Millipore, Darmstadt, Germany). For total Erk1/2, after detection of phosphorylated Erk1/2, the same membrane was washed with stripping buffer (46428, Thermofisher, United States) for 30 min, and the subsequent steps were the same as described above.

### Enzyme-linked immunosorbent assay kit

Serum samples from mice and supernatant of cell culture were subjected to measurement of irisin and Mstn using enzyme-linked immunosorbent assay (ELISA). The assays were conducted according to the procedures recommended in the manufacturer’s instructions of irisin ELISA kits (AG-45A-0046YEK-KI01, Adipogen, LIFE SCIENCE, Missouri, United States) and Mstn ELISA kits (CSB-EL015057MO, R&D Systems, CUSABIO, China).

### Statistical analysis

Data were shown as mean ± SD. Unpaired Student’s t-test was performed to evaluate statistical differences between two groups. For multiple comparisons, data were analyzed using one-way analysis of variance (ANOVA) with SPSS 20.0 software (IBM Corporation, Armonk, NY, United States). If equal variances were assumed, the Tukey *post hoc* test was selected, and if not, Dunnett’s T3 was selected. A value of *p* < 0.05 was considered significant statistically.

## Results

### Long-term cigarette smoke exposure led to skeletal muscle dysfunction

**FIGURE 1:**
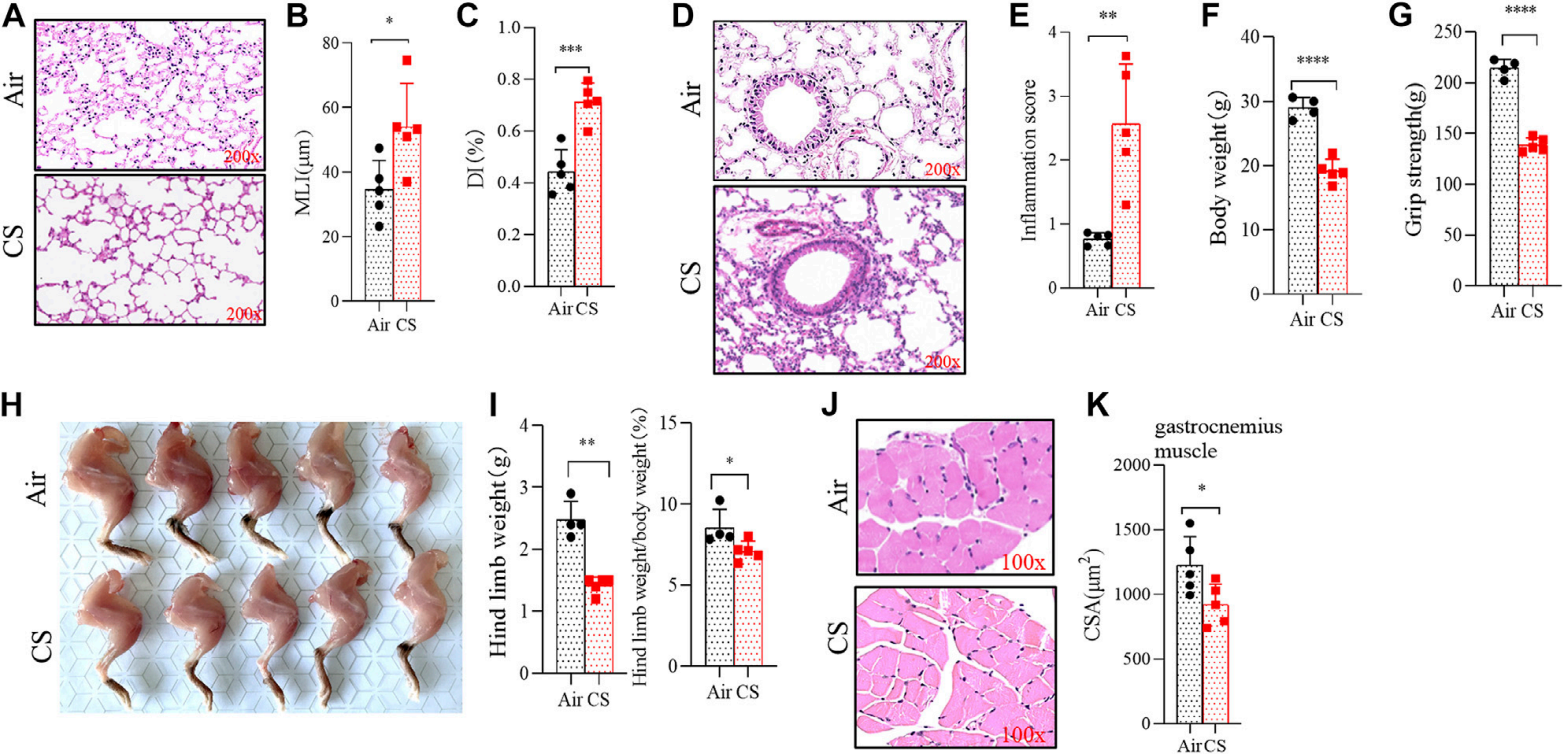
Long-term cigarette smoke (CS) exposure led to skeletal muscles dysfunction. **(A,D)** hematoxylin–eosin (H&E) staining of lung tissues from mice exposed to air and CS. **(B,C)** histogram of mean linear intercept and destructive index. **(E–G)** histogram of inflammation score, body weight, and grip strength. **(H)** anatomic morphology of hind limbs. **(I)** statistical results of hind limbs weight (left) and hind limbs weight/body weight (right). **(J)** H&E staining of the gastrocnemius muscle. **(K)** statistical results of the mean cross-sectional area of gastrocnemius muscle fibers. *n* = 5, *p < 0.05, **p < 0.01, ***p < 0.001, ****p < 0.0001.

Mice exposed to CS for 24 weeks exhibited alveolar space enlargement (Figure 1A), the MLI and DI being significantly increased in CS-exposed mice (Figures 1B, C). Significant inflammatory infiltration was also observed in lung tissues of CS-exposed mice (Figures 1D, E). These changes were consistent with COPD. It was worth noting that the loss of body weight was associated with changes in muscle mass (Figure 1F), and the grip force test showed that CS-exposed mice had a significant decrease in grip strength (Figure 1G). In addition, the morphology study showed a significant reduction in the hind limb muscle mass in CS-exposed mice (Figures 1H, I). The CSA of the gastrocnemius muscle from CS-exposed mice was markedly decreased, and the muscle texture was looser than that of the control group (Figures 1J, K).

### Neurodegeneration, mitogen-activated protein kinase, and ubiquitin-proteasome signaling pathways were upregulated in skeletal muscles of chronic obstructive pulmonary disease mice

**FIGURE 2:**
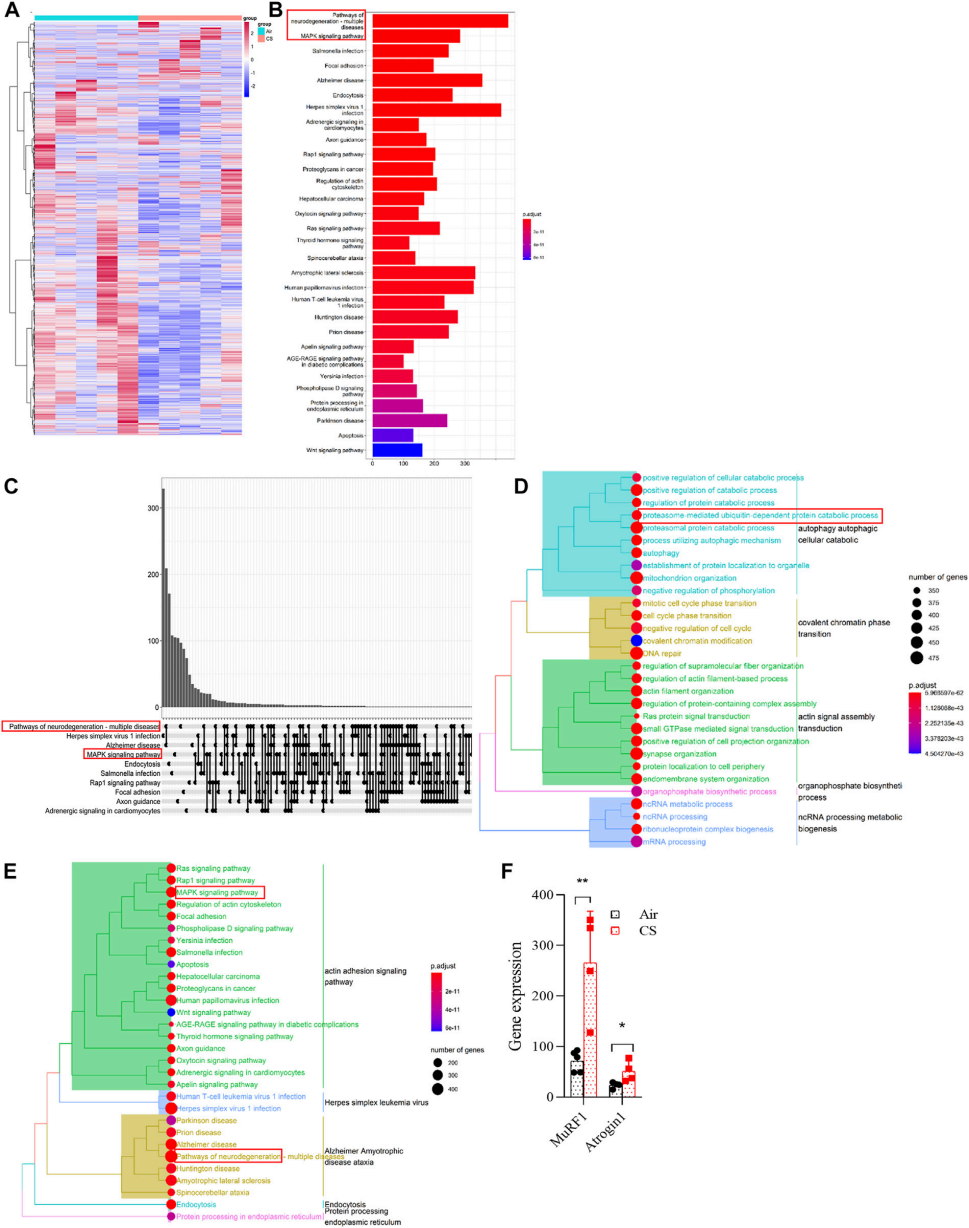
Neurodegeneration, MAPK, and ubiquitin-proteasome signaling pathways were upregulated in skeletal muscles of chronic obstructive pulmonary disease mice. **(A)** heatmap showing the enrichment of genes in gastrocnemius muscle by RNA sequencing. **(B,C)** histogram and upset plot showing the enrichment results of KEGG. **(D)** GO treeplot showing the different signaling pathways using average expression of differential genes in the gastrocnemius muscle. **(E)** KEGG treeplot showing the different signaling pathways using average expression of differential genes in the gastrocnemius muscle. **(F)** gene expression of *MuRF1* and *Atrogin1* in RNA-seq. *n* = 5, *p < 0.05, **p < 0.01.

In order to reveal the dysregulated genes in skeletal muscles from COPD mice, we first performed RNA sequencing and found a significant enrichment of the transcriptome (Figure 2A). The enrichment of signal pathways using KEGG showed that neurodegeneration, mitogen-activated protein kinase (MAPK), and ubiquitin-proteasome signaling pathways were significantly upregulated in CS-exposed mice (Figures 2B–E), which provided clues to our subsequent mechanism investigation. MuRF1 and Atrogin1 are important ubiquitin ligases in the ubiquitin-proteasome pathway that mediate muscle proteolysis, and here, we found that their corresponding gene expressions of *MuRF1* and *Atrogin1* were significantly upregulated in skeletal muscles of COPD mice (Figure 2F).

### Cigarette smoke exposure attenuated fibronectin type III domain-containing protein 5/irisin production but augmented myostatin expression in skeletal muscles

**FIGURE 3:**
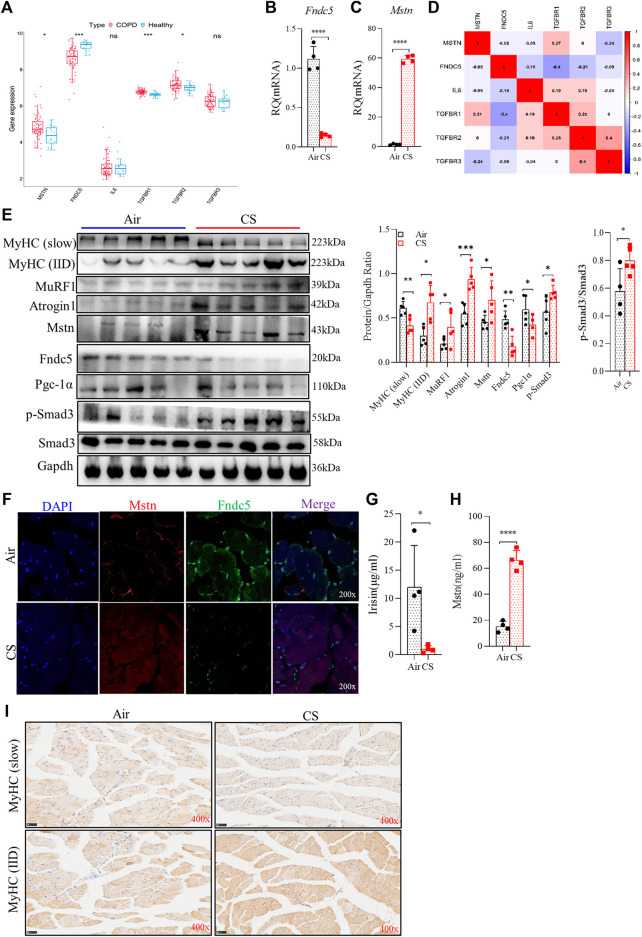
Cigarette smoke exposure attenuated fibronectin type III domain-containing protein 5 (Fndc5)/irisin production but augment myostatin (Mstn) expression in skeletal muscles and serum, accompanied by muscle fiber-type switch. **(A)** boxplot of the statistical results of some myokines and related receptors, such as *Mstn, Fndc5, IL-6, TGF-βR1, TGF-βR2*, and *TGF-βR3*. **(B,C)** real-time quantitative PCR examined the expression of Fndc5 and Mstn from gastrocnemius muscle. **(D)** corplot showing the correlation between Fndc5 and TGF-βR1. **(E)** WB explored the protein production (left), the ratio of protein to Gapdh (middle), and the ratio of p-Smad3 to Smad3 (right). **(F)** expression of Fndc5 and Mstn distributed in the gastrocnemius muscle using confocal microscopy. **(G,H)** levels of irisin and Mstn in serum were detected using ELISA. **(I)** expression of MyHC (slow) and MyHC (IID) were explored using immunohistochemistry. *n* = 5, *p < 0.05, **p < 0.01, ***p < 0.001, ****p < 0.0001, ns: not significant.

Mstn is a well-recognized negative regulator of muscle growth, whereas Fndc5 is a positive one in skeletal muscles (Cohen et al., 2015; Reza et al., 2017). As important myokines, Mstn and Fndc5/irisin play opposite roles in skeletal muscles and can be used as indicators of muscle function (Planella-Farrugia et al., 2019). Here, we explored the expression of Mstn, Fndc5, and their upstream and downstream signaling molecules. RNA-seq and real-time quantitative PCR (RT-qPCR) analysis showed that elevated *Mstn* and decreased *Fndc5* were observed at gene levels in the quadriceps femoris of patients with COPD from GEO (GSE 100281) (Willis-Owen et al., 2018) (Figure 3A) and in the gastrocnemius muscle from CS-exposed mice (Figures 3B,C). Moreover, the level of *Fndc5* was negatively correlated with that of *TGF-βR1* (also known as ALK5) (Figure 3D). Protein levels of MuRF1, Atrogin1, Mstn, and Fndc5 displayed consistent trends with gene levels in the gastrocnemius muscle (Figure 3E). Both p-Smad3 and PGC-1α are the upstream regulators of Fndc5. To investigate whether the Smad3/PGC-1α signaling pathway was affected by CS exposure, we detected p-Smad3 and PGC-1α in the gastrocnemius muscle simultaneously. Western blot analysis showed that PGC-1α was decreased, while p-Smad3 was increased in skeletal muscles from CS-exposed mice (Figure 3E), indicating that the Smad3/PGC-1α signaling pathway was activated. Of note, we found that the protein levels of muscle-specific E3-ubiquitin ligases MuRF1 and Atrogin1 associated with muscle proteolysis were significantly increased in muscles from CS-exposed mice (Figure 3E). We also observed the expression of Mstn and Fndc5 in skeletal muscle cells by confocal IF, which showed that Mstn was increased, while Fndc5 was decreased in CS-exposed mice compared with the air-exposed ones (Figure 3F). Fndc5 is a transmembrane precursor protein expressed in muscles, and irisin is cleaved from Fndc5 and released into blood. Therefore, we further detected the circulating levels of irisin and Mstn, and we found that serum irisin was significantly reduced, while that of Mstn was increased in CS-exposed mice (Figures 3G, H). In addition, we also examined muscle fiber-type switch in the gastrocnemius muscles from the mice, and we found that the expression of MyHC of the slow-twitch fibers was significantly decreased, while that of the fast-twitch fibers (IID) was significantly enhanced, in the CS-exposed group, which may indicate that there may be a slow-to-fast-fiber-type switch in this process (Figures 3E, I).

### Cigarette smoke extract altered myostatin and fibronectin type III domain-containing protein 5 expression in C2C12 myotubes

**FIGURE 4:**
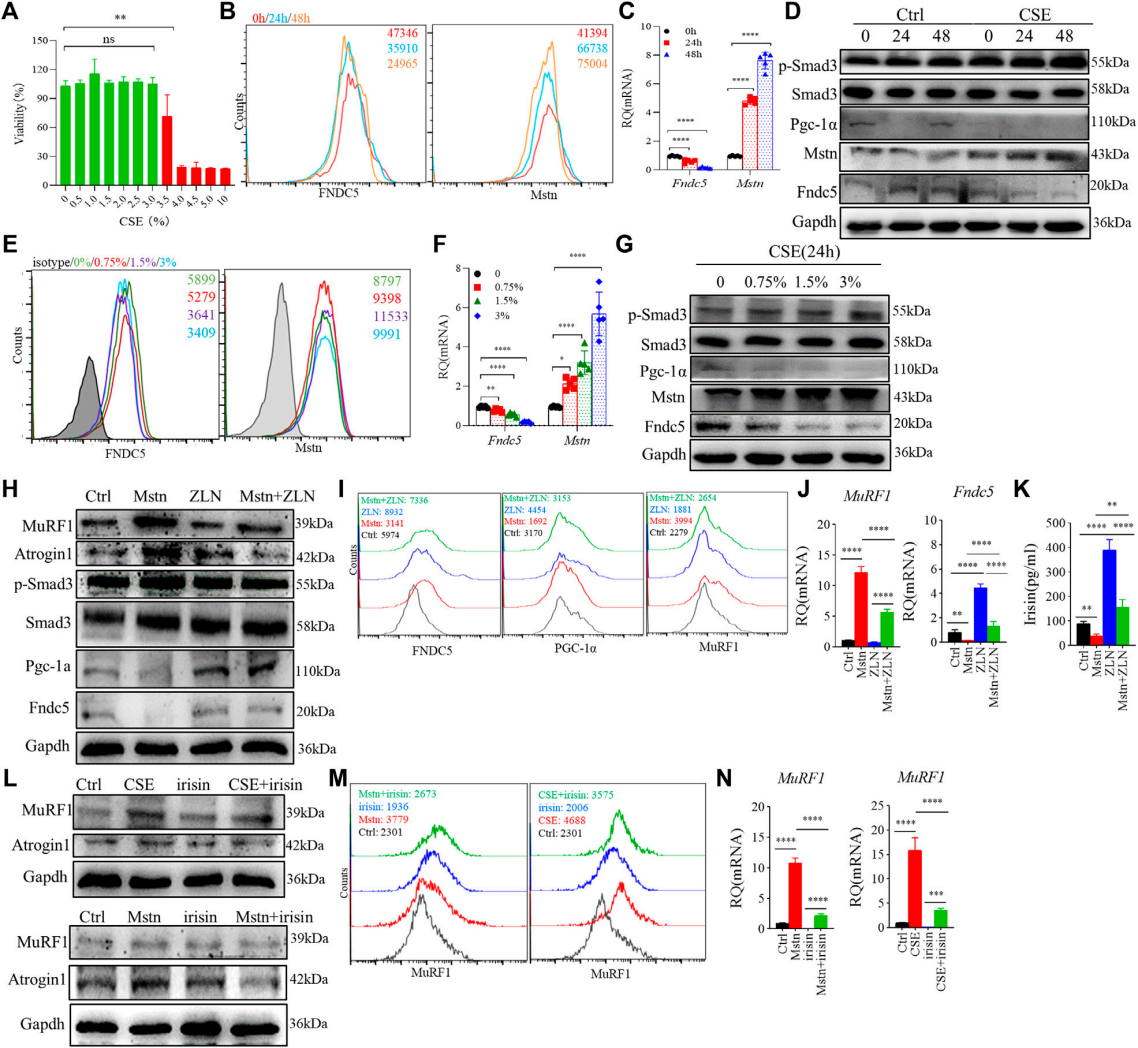
Cigarette smoke extract (CSE) altered myostatin (Mstn) and Fndc5 expression in C2C12 myotubes. **(A)** cell viability of C2C12 myotubes was tested in Dulbecco’s modified Eagle’s medium containing 10% fetal bovine serum and stimulated with different concentrations of CSE for 24 h. **(B)** histogram showing the mean fluorescence intensity (MFI) of fibronectin type III domain-containing protein 5 (Fndc5) and myostatin (Mstn) in 3% CSE-stimulated C2C12 myotubes at indicated time points. **(C)** expression of *Fndc5* and *Mstn* in 3% CSE-stimulated C2C12 myotubes at indicated time points were detected using real-time quantitative PCR (RT-qPCR). **(D)** WB showing the expression of different proteins in 3% CSE-stimulated C2C12 myotubes. **(E)** histogram showing the MFI of Fndc5 and Mstn in CSE-stimulated C2C12 myotubes at different concentrations of CSE. **(F)** expression of *Fndc5* and *Mstn* in CSE-stimulated C2C12 myotubes at different concentrations of CSE were detected using real-time quantitative PCR. **(G)** WB showing the expression of different proteins in CSE-stimulated C2C12 myotubes at different concentrations of CSE for 24 h. **(H)** WB showing the expression of different proteins in Mstn and/or ZLN005 (ZLN, 10 μM)-stimulated C2C12 myotubes for 24 h, which also be detected using FACS **(I)** and RΤ-qPCR **(J)**. **(K)** supernatant of cell culture was detected using ELISA. **(L)** WB showing the expression of different proteins in recombinant irisin (100 ng/ml, up), Mstn (100 ng/ml, down), and/or 3% CSE-stimulated C2C12 myotubes for 24 h, which were also detected using FACS **(M)** and RΤ-qPCR **(N)**. *n* = 3, *p < 0.05, **p < 0.01, ***p < 0.001, ****p < 0.0001, ns: not significant.

We used CSE-stimulated C2C12 myotubes to further explore the effect of CS exposure on myokine expression and related signaling molecules. First, we confirmed the optimal dose of CSE (<3%) for subsequent experiments using the CCK-8 method, which reflected the cell viability (Figure 4A). In the present study, we used 3% CSE to stimulate C2C12 myotubes for 24 and 48 h. The obtained myotubes were directly stimulated by CSE *in vitro*, and flow cytometry analysis showed that the mean fluorescence intensity (MFI) of Fndc5 decreased significantly in a certain time range, while that of Mstn was increased (Figure 4B). Consistent with the above findings, the mRNA levels of *Fndc5* were reduced while those of *Mstn* increased after CSE exposure (Figure 4C). Western blotting of cell homogenates confirmed these findings (Figure 4D). To observe the effects of CSE stimulation on Smad3/PGC-1α signaling pathway *in vitro*, we also detected p-Smad3 and PGC-1α in cultured cells, which showed decreased PGC-1α but increased p-Smad3 levels in CSE-stimulated myotubes (Figure 4D). To confirm the dose response of CSE on C2C12 myotubes, we used different dose concentrations of CSE (0, 0.75%, 1.5%, 3%) to stimulate C2C12 myotubes for 24 h. The results showed that the protein and mRNA levels of Fndc5 were inhibited, while those of Mstn were enhanced with increase of CSE concentration (Figures 4E–G). The changes of PGC-1α and p-Smad3 were consistent with the above results (Figure 4G).

Next, we further investigated whether the elevated Mstn have direct effects on skeletal muscle cells. We treated C2C12 myotubes with recombinant Mstn *in vitro* and found that MuRF1 and Atrogin1 were significantly enhanced, accompanied by upregulation of downstream signaling molecules p-Smad3, but PGC-1α and Fndc5 were significantly decreased, suggesting that the Mstn-Smad3 pathway could inhibit PGC-1α/Fndc5 production. It is reported that ZLN005 is a potent activator of PGC-1α by activating AMP-activated protein kinase (AMPK) in a dose-dependent manner. After treatment with ZLN005, we found that the upregulated expression of PGC-1α and the production of Fndc5 and irisin were restored (Figures 4H–K). In addition, we also explored the direct effects of irisin on C2C12 myotubes, and we found that irisin did partially alleviate the harm effects of CSE or Mstn on C2C12 myotubes, showing the decreased level of MuRF1 and Atrogin1 (Figures 4L–N).

### Cigarette smoke extract exposure led to skeletal muscle dysfunction through multiple mechanisms in C2C12 myotubes

**FIGURE 5:**
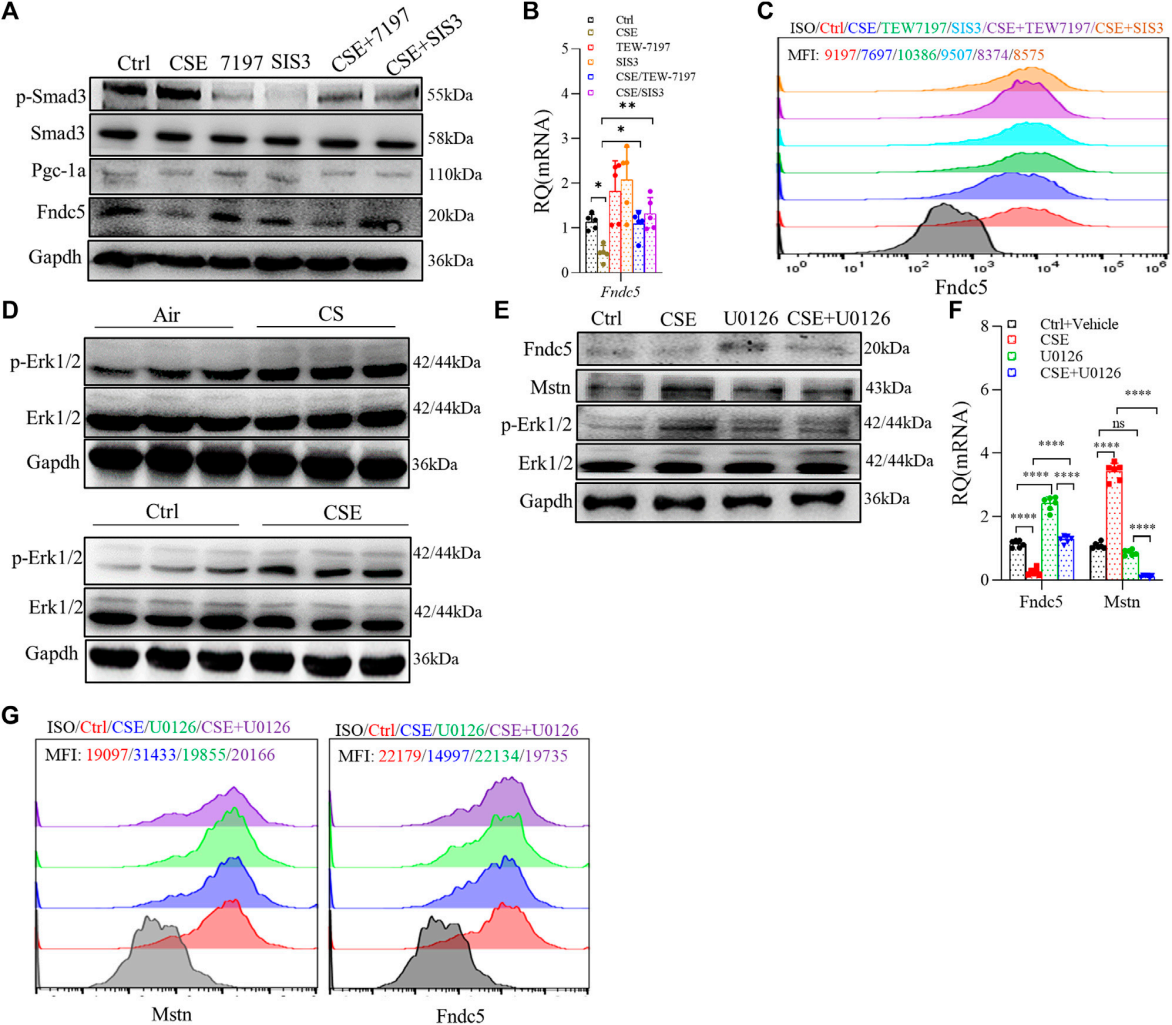
Cigarette smoke extract (CSE) exposure led to skeletal muscle atrophy through multiple mechanisms in C2C12 myotubes. C2C12 myotubes were treated with 3% CSE, TEW-7197 (10 μM), and SIS3 (10 μM) for 24 h and then examined using WB **(A)**, real-time quantitative PCR (RT-qPCR) **(B),** and FACS **(C)**. p-Erk1/2 was examined in the gastrocnemius muscle from cigarette smoke (CS)- and air-exposed mice and in C2C12 myotubes stimulated with 3% CSE **(D)**. C2C12 myotubes were treated with 3% CSE and U0126 (5 μM) for 24 h and then examined using WB **(E)**, RT-qPCR **(F)**, and FACS **(G)**. mean fluorescence intensity is shown using the same indicated colors **(C,G)** (n = 3, *p < 0.05, **p < 0.01, ****p < 0.0001, ns: not significant).

As mentioned above, the Smad3/PGC-1α pathway played an important role in regulating Fndc5 expression, so we investigated the Smad3/PGC-1α pathway by using TEW-7197 (ALK4/5 inhihitor) and SIS3 (Smad3 inhibitor), and we found that Fndc5 production was partially restored in C2C12 myotubes treated with 3% CSE after inhibition of Smad3 (Figures 5A–C). Based on our RNA-seq data that MAPK was involved in skeletal muscle dysfunction (Figures 2B, C, E), we hypothesized that Erk1/2 could be one of the proteins dysregulated in the CSE-treated C2C12 myotubes, which was verified by blocking experiments using U0126, a specific inhibitor of Erk1/2. First, the expression of p-Erk1/2 was significantly upregulated in both skeletal muscles from COPD mice and CSE-stimulated C2C12 myotubes (Figure 5D). Second, we also found that Mstn production was downregulated in CSE-stimulated C2C12 myotubes after U0126 treatment, while the expression of Fndc5 was partly recovered with the downregulation of Mstn (Figures 5E–G), suggesting an interaction between these two myokines in this model.

## Discussion

Although it has been reported that the dysregulation of Mstn and irisin is involved in skeletal muscle dysfunction in CS-exposed models or in patients with COPD, the molecular pathways regulating these myokines in COPD remain elusive. In the current study, by using a well-established model of COPD with skeletal muscle dysfunction, we found that the expression of Fndc5 was downregulated, with reduced PGC-1α but increased p-Smad3 expression, in skeletal muscles from CS-exposed mice and in CSE-stimulated C2C12 myotubes, accompanied by enhanced expression of Mstn. We found that CSE promoted Mstn expression by enhancing p-Erk1/2, and Mstn further inhibited Fndc5 expression by the p-Smad3/PGC-1α pathway, which may serve as a mechanism for accelerated development of skeletal muscle dysfunction in COPD. To our knowledge, this is the first study to show that the p-Erk1/2 and the Smad3/PGC-1α signaling pathway are involved in the downregulation of Fndc5/irisin in COPD-related skeletal muscle dysfunction.

Mstn is primarily derived from skeletal muscles and is a potent negative regulator of muscle function (McPherron et al., 1997). There was an inverse correlation between the expression of Mstn mRNA in muscles and insulin sensitivity, and both acute and long-term exercise downregulated the expression of Mstn in skeletal muscles (Hjorth et al., 2016). Evidence supports the involvement of Mstn in the muscle atrophy of COPD. For example, cigarette smoking increased the expression of Mstn in muscles (Petersen et al., 2007), and Mstn was upregulated in the vastus lateralis muscle of COPD patients (Plant et al., 2010) and in the diaphragm of COPD rats (Zhou et al., 2018). In our study, we also found upregulated Mstn in CS-exposed mice and CSE-stimulated C2C12 myotubes, which was consistent with previous reports.

In contrast to the consistent findings with Mstn in many literatures, studies on the newer myokine-irisin were limited, and the results were varied in COPD. One study showed that the serum level of irisin was decreased and associated with physical activity level in COPD patients (Ijiri et al., 2015). Another study found that the serum level of irisin was negatively associated with the percentage of low-attenuation areas (%LAA) and diffusing capacity of the lungs for carbon monoxide divided by the alveolar volume (DL_CO_/V_A_) in COPD (Sugiyama et al., 2017). Both DL_CO_/V_A_ and %LAA are markers for the severity of emphysema. An interesting study found that irisin enhanced the expression of nuclear factor erythroid 2-related factor 2 (Nrf2), a critical regulator of the antioxidant system, and reduced CSE-induced A549 cell apoptosis (Sugiyama et al., 2017), which indicated that irisin may play a protective role in cell apoptosis by way of antioxidation. However, the specific molecular mechanisms underlying the dysregulation of Fndc5 in skeletal muscles in COPD are not clear. Here, we examined the expression of Fndc5 in skeletal muscles of COPD mice and found that Fndc5/irisin production was markedly reduced. CSE exposure inhibited Fndc5 expression in C2C12 myotubes *in vitro.* Irisin promotes skeletal muscle growth through enhancing satellite cell activation and decreasing protein degradation. The decreased Fndc5/irisin levels in COPD may impair skeletal muscle growth and developmental potential and weaken the antioxidative capacity, thereby promoting muscle atrophy and dysfunction. In addition, our studies demonstrated that recombinant irisin partially alleviated the harmful effects of CSE or Mstn on C2C12 myotubes.

Although it is known that Mstn inhibits PGC-1α and thus Fndc5 expression by activating p-Smad3 (Dong et al., 2016), the interaction between these two myokines in the pathogenesis of skeletal muscle dysfunction in COPD has not been reported. Here, we detected some key molecules (p-Smad3 and PGC-1α) involved in the irisin and Smad3 signaling pathway (Shan et al., 2013; Tiano et al., 2015). Our data showed that the production of PGC-1α in skeletal muscle cells was downregulated, while Smad3, the upstream negative regulator, was upregulated *in vivo* and *in vitro*. In addition, bioinformatic analysis showed that the mRNA level of *TGF-βR1* was enhanced and negatively associated with *Fndc5* in the quadriceps of COPD patients. In the *in vitro* model, we used two inhibitors, TEW-7197, a selective TGF-β receptor ALK4/ALK5 inhibitor that prevents Mstn binding to its receptor, and SIS3, a specific Smad3 inhibitor by inhibiting Smad3 phosphorylation, to suppress the Smad3 signaling pathway. We hypothesized that if CSE could directly affect the expression of Fndc5, ALK4/ALK5 inhibition alone with TEW-7197 could only partially restore the expression of Fndc5, and if CSE inhibited the expression of Fndc5 only by Smad3, Fndc5 production could be completely restored by directly inhibiting Smad3 with SIS3. Our study found that both mechanisms were involved; that is, on the one hand, CSE inhibited Fndc5 expression by promoting Mstn expression, and on the other hand, CSE inhibited Fndc5 production directly or through other pathways.

Based on our RNA sequencing results, we found that MAPK may be involved in skeletal muscle dysfunction of COPD. We found that p-Erk1/2 was upregulated after CSE stimulation, while Mstn expression was reduced after Erk1/2 inhibition by U0126, and Fndc5 exhibited a certain degree of recovery with the downregulation of Mstn, suggesting that CSE upregulated Mstn expression through the Erk1/2 pathway.

There were several limitations to our study. First, Fndc5/irisin was regulated by exercise, but we did not implement exercise intervention on mice to study the changes of myokine expression induced by exercise. Second, we did not examine the direct effects of irisin on skeletal muscle growth and function *in vivo*, and therefore, a relationship could not be well established between decreased irisin expression and impaired skeletal muscle function. Further studies are needed to delineate the exact role of irisin and related signaling pathways in skeletal muscle dysfunction/sarcopenia in COPD.

## Conclusion

**FIGURE 6:**
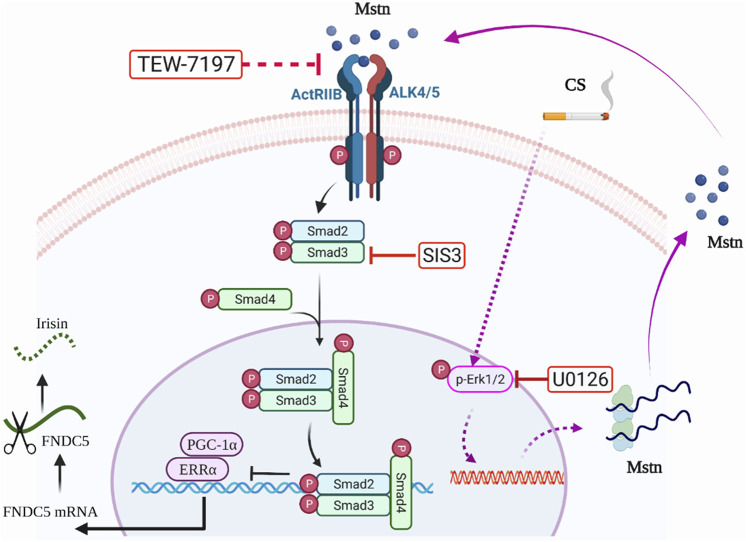
Graphic summary. On the one hand, cigarette smoke extract (CSE) exposure could enhance myostatin (Mstn) production through the Erk1/2 pathway, which further activated the Smad3/PGC-1α pathway, and negatively regulated Fndc5 production. On the other hand, CSE exposure might partially and directly decrease the expression of Fndc5. TEW-7197, a selective TGF-β receptor ALK4/ALK5 inhibitor that can stop Mstn binding to its receptor. SIS3, a specific Smad3 inhibitor by inhibiting Smad3 phosphorylation to suppress Smad3 signaling pathway. U0126, a specific inhibitor of Erk1/2.

In conclusion, we found dysregulated expression of the myokines-irisin and Mstn in a long-term CS exposure-induced mouse model of COPD accompanied by impaired skeletal muscle mass and strength. We also demonstrated that CSE led to an increase in Mstn expression by enhanced p-Erk1/2, and Mstn further inhibited Fndc5/irisin expression by the p-Smad3/PGC-1α pathway, which may play a critical role in accelerating the development of skeletal muscle dysfunction in COPD, revealing a novel regulating mechanism of myokines in the pathogenesis of the skeletal muscle comorbidity of COPD (Figure 6). More studies are warranted to further dissect the roles of irisin and related signaling pathways in skeletal muscle dysfunction in COPD.

## Data Availability

The accession number of the RNA-seq data reported in this article is GEO: GSE197463. The raw data supporting the conclusions of this article will be made available by the authors, without undue reservation.
